# Care of Teeth

**Published:** 1887-01

**Authors:** 


### CARE OF TEETH.

A mouthful of good teeth is one of the rare gifts of nature. Like bright eyes, pink-mooned finger nails, or a fine complexion, they indicate the bequests of heredity, and are symbolic of a sweet breath, good digestion and a wholesome stomach. A wealth of dentine is not as highly prized as formerly, owing to the remarkable progress made in dentistry within the last quarter of a century. Molar-menders think nothing of working a *cheval de frise* sort of a set of teeth into a double row of presentable ivories, and the skill with which china teeth are made to duplicate nature is sufficient to keep the genuine articles under a constant ban of suspicion. All these facts were doubtless known to the fashionable mother who prayed for "just such eyes and fine complexion" for her little daughter. Eyes and skin from nature, and art can manage the rest, at least to the satisfaction of the modern beauty.

In remodeling teeth, everything fails before the final surrender to a false set. Where they overlap, space has to be made at the sacrifice often of good material, and when by accident, a tooth is wanted, the gap is filled by spacing the whole row.

In the color of teeth almost as much variety exists as in hair and eyes. Some teeth are naturally gray, yellowish or blueish in cast, and to try and whiten them is time wasted. The only solace lies in keeping them clean and straight. It is immaterial to any one with a moustache or a very long upper lip, whether he has any front teeth or not. For ladies or beardless men, especially those who langh much with the lips, a remedy is sought among the Del s'Arte people. These refiners of nature attempt, and with success, too, to cultivate a very low voice in speaking, forbid the license of heated discussion, and endeavor to cultivate a laugh in the eyes, rather than about the lips. The training is a long and tedious task, but there are few ordeals too severe for a fashionable man or woman to endure, when the goal is good looks.

It is almost impossible to say anything new on the subject of powders. The best powder is the one that does the least harm to the gums and keeps the enamel clean. Wintergreen is safe to any polish, but a frequent use of soft soap and warm water renders much of the cleansing powder superfluous. Teeth that are brushed four times a day will not need a powder more than once a week. Tooth-picks are indispensable, and even with them it is often necessary to run a thread between the teeth to remove any possible accumulation or splinter.

There might be a diminution of dentistry bills if those who have teeth would take the trouble to clean them once a month. Five cents' worth of pumice stone will cover a year, and nothing but a match is needed to start with. Dip the pine in the stone and rub between the teeth till all trace of mineral accumulation has been removed. The inside surface must be cleaned separately, and the task finished by rubbing the face and crown of the tooth with a soft handkerchief dipped in the powder. Unless the operation is made habitual it will consume the best part of an hour to produce any good effects.—*Ex.*

